# Adult Patient with Synchronous Gastrointestinal Stromal Tumor and Xp11 Translocation-Associated Renal Cell Carcinoma: A Unique Case Presentation with Discussion and Review of Literature

**DOI:** 10.1155/2015/814809

**Published:** 2015-07-13

**Authors:** Vanda Farahmand Torous, Albert Su, David Y. Lu, Sarah M. Dry

**Affiliations:** ^1^Department of Pathology and Laboratory Medicine, Beth Israel Deaconess Medical Center, 330 Brookline Avenue, Boston, MA 02215, USA; ^2^Department of Pathology and Laboratory Medicine, David Geffen School of Medicine at UCLA, 10833 Le Conte Avenue, 13-145G CHS, Los Angeles, CA 90095-1732, USA

## Abstract

Gastrointestinal stromal tumor (GIST) is the most common primary mesenchymal tumor of the gastrointestinal tract. This entity comprises a wide spectrum of tumors that vary from benign to overtly malignant, with the majority of these tumors harboring oncogenic mutations of the KIT receptor tyrosine kinase that can aid in diagnosis as well as in targeted therapy. Although the majority of GISTs are sporadic, there are forms that are associated with a variety of syndromes including Carney-Stratakis syndrome and neurofibromatosis type 1, as well as a subset of familial GIST syndromes that are caused by germline mutations in KIT or PDGFRA. Here, we describe an unusual case of a patient who was found to have a large abdominal GIST with an incidentally found Xp11 translocation-associated renal carcinoma. The karyotype of the renal carcinoma revealed an unbalanced rearrangement involving an (X;22) translocation at Xp11.2 and 22p11.2, which has not been reported in the literature. Although GISTs have shown an association with other primary malignant neoplasms, including simultaneous presence with unilateral clear cell renal cell carcinoma and bilateral papillary renal cell carcinomas, we describe the first reported case of synchronous GIST and Xp11 translocation-associated renal cell carcinoma.

## 1. Introduction

Gastrointestinal stromal tumor (GIST) is the most common primary mesenchymal tumor in the GI tract. The true frequency of GIST has been difficult to determine because it was not molecularly characterized until recently, although some population-based studies have suggested an annual incidence of 11–15 per million population [1]. The majority of GISTs appear to occur sporadically. However, about 5 percent of GISTs are associated with syndromes or specific inheritable mutations.

We report a case of a patient with a large gastric GIST and an incidentally found Xp11 translocation-associated renal carcinoma, which harbored a previously unreported (X;22) translocation involving Xp11.2 and 22p11.2. Although GISTs have been reported to show an association with other primary malignancies, including renal cell carcinoma (RCC), this is the first reported case of GIST occurring synchronously with an Xp11 translocation-associated renal carcinoma.

## 2. Case Presentation

A 66-year-old female with a past medical history of hypertension, hypothyroidism, and gastroesophageal reflux disease presented to an outside hospital with abdominal bloating and discomfort. An ultrasound performed at her initial presentation noted a large left upper abdominal mass. She was referred for a whole body positron emission tomography-computed tomography (PET-CT) scan that demonstrated a large 24 × 12 cm left upper abdominal tumor coming off the tail of the pancreas and abutting the greater curvature of the stomach. The patient also had hypermetabolic metastases within the liver.

The patient underwent an image-guided biopsy of the large lesion. Pathology demonstrated a bland spindle cell neoplasm consistent with GIST. Immunohistochemistry was positive for CD117 and CD34. One-two mitoses were identified on the entire core tissue, and Ki-67 showed 1-2% proliferative index. The patient was started on imatinib (Gleevec) and demonstrated a metabolic response to therapy with a slight decrease in the size of the tumor.

**Figure 1:**
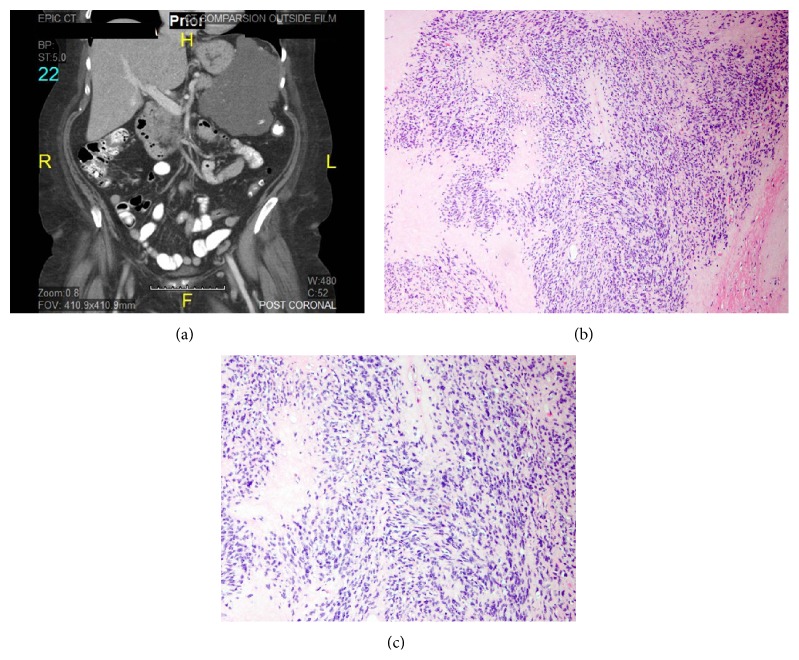
(a) Computed tomography (CT) demonstrated a large, 15 cm left upper abdominal tumor stemming from the wall of the stomach. Multiple hepatic lesions consistent with metastatic tumor were also identified. (b) Microscopically, scant areas of viable tumor are identified in the patient's GIST (patient after imatinib therapy). (c) Viable tumor was composed of elongated spindle-shaped cells with vesicular chromatin and abundant cytoplasm arranged in fascicles and sheets.

Four months after the initiation of imatinib therapy, a follow-up CT demonstrated the prior GIST, which had decreased in size to 13.2 × 8.9 × 12.9 cm (Figure 1(a)). Multiple hepatic lesions were once again identified, although most of them had decreased attenuation and showed a decrease in size. However, a 2.8 × 2.6 × 1.9 cm ovoid, mixed density, and partially calcified left kidney mass in the mid to lower pole was also identified, radiographically consistent with a primary RCC.

Given that the patient had an excellent radiographic and clinical response to imatinib and an enlarging left renal tumor that was radiographically concerning a primary RCC, resection of both the gastric and renal masses was recommended. The patient underwent a simultaneous radical resection of the large upper abdominal mass, consisting of en bloc subtotal gastrectomy, distal pancreatectomy, and partial omentectomy, as well as left radical nephrectomy, 10 months after initial presentation.

Grossly, a 14.7 × 8.7 × 7.3 cm large mass stemming from the wall of the stomach was identified. The mass appeared tan-gray and lobulated on cut resection surfaces, with scattered edematous areas. Microscopically, the tumor demonstrated greater than 95% necrosis, consistent with the radiographic findings of response to imatinib. Scant areas demonstrated viable spindle-shaped tumor cells with vesicular chromatin and abundant cytoplasm arranged in fascicles and sheets, consistent with a GIST. The mitotic rate was low, with 0 mitoses identified per 5 mm^2^ (0-1 mitoses per 10 high power fields) (Figures 1(b) and 1(c)). Gene mutation testing showed a KIT exon 11 deletion/substitution KPMYEV 550–555 L. PDGFRA mutation testing was not performed.

**Figure 2:**
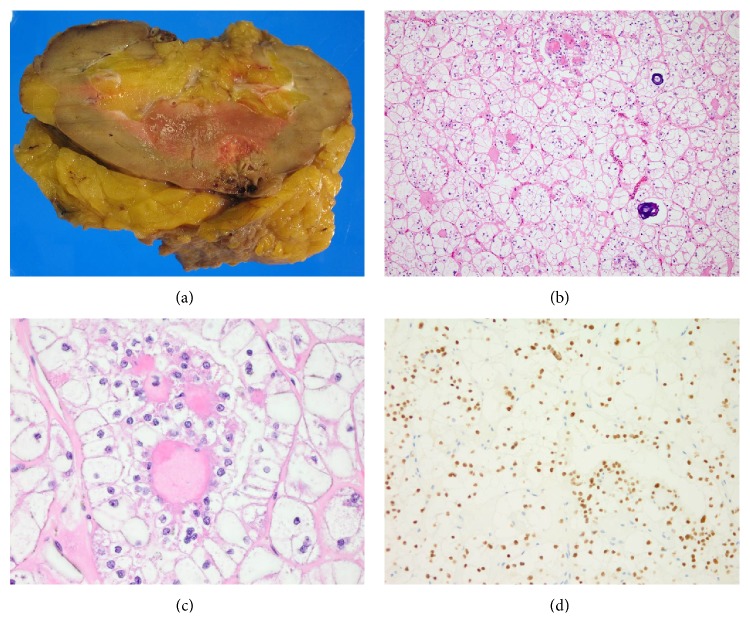
(a) Gross image of the nephrectomy specimen shows a 3.6 cm hemorrhagic mass in the lower renal pole. The mass abuts the renal sinus fat. (b) Microscopically, the tumor consists of variably sized nests of cells that are separated by fibrovascular septa. Psammomatous calcifications and focal rounded hyalinized structures are present. (c) Tumor cells have abundant clear to focally eosinophilic granular cytoplasm. (d) The tumor was found to be TFE3 positive.

**Figure 3:**
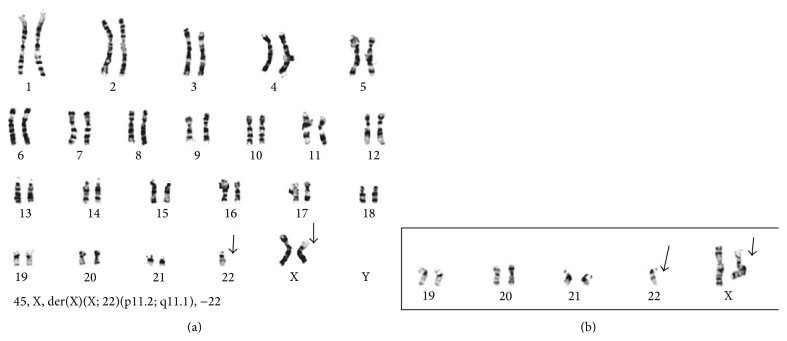
This is an unbalanced translocation between Xp and 22q resulting in a loss of Xp and 22p regions shown in (a) complete metaphase and in (b) a partial metaphase.

The nephrectomy specimen demonstrated a 3.6 × 3.2 × 1.8 cm hemorrhagic mass in the lower renal pole that abutted the renal sinus fat and came within 0.2 cm of the perinephric fat (Figure 2(a)). On hematoxylin and eosin- (H&E-) stained sections, the renal mass was focally well-circumscribed, though in a few areas it exhibited tumor extensions into adjacent parenchyma. It consisted of variably sized nests of cells separated by thin, fibrovascular septa (Figure 2(b)). The neoplastic cells were round to polygonal and demonstrated mainly voluminous clear to focally eosinophilic granular cytoplasm, distinct cell membranes, and mildly to moderately atypical vesicular nuclei with occasional nucleoli (Figure 2(c)). Some of the cells were binucleated to trinucleated. In several areas, the tumor nests demonstrated one to multiple rounded hyalinized structures. These hyalinized foci were usually lined by cells with higher nuclear-to-cytoplasmic ratio than, but similar nuclei to, the large cells comprising the majority of the tumor nests. Within scattered tumor nests, there was cellular dyscohesion away from the hyalinized foci with only the layer of small cells remaining, imparting a focal papillary or pseudopapillary appearance. Abundant psammomatous calcifications were seen throughout, often within the hyalinized areas. The tumor cells were positive by immunohistochemistry for vimentin, MART-1/Melan A, and the renal tubular marker CD10 (scattered focal membranous positive). They showed strong and diffuse nuclear positivity with transcription factor E3 (TFE3) (Figure 2(d)), and they were negative for cytokeratin AE1/AE3, epithelial membrane antigen (EMA), cytokeratin 7, carbonic anhydrase 9 (CA9), and HMB-45. Cytogenetic studies performed on fresh tissue from the tumor demonstrated an unbalanced rearrangement characterized by an (X;22) translocation at Xp11.2 and 22p11.1, consistent with an Xp11.2 (*TFE3* gene) translocation-associated renal carcinoma (Figure 3). As the tumor invaded the renal sinus fat, it was classified as pathologic TNM stage pT3a pNX MX, or Stage III.

On six-month follow-up, the patient was doing well. She was continuing on imatinib therapy due to the metastatic disease involving her liver, which also demonstrated the same KIT exon 11 mutation as her primary GIST.

## 3. Discussion

Although most GISTs are sporadic, approximately 5% occur in association with various syndromes including Carney triad (GIST, paraganglioma, and pulmonary chondroma), Carney-Stratakis syndrome (GIST, paraganglioma), and neurofibromatosis type 1 (NF1). Specific inheritable gene mutations have also been described in families predisposed to develop GISTs; these include mutations of KIT, PDGFRA, and succinate dehydrogenase genes [2–13]. GISTs have also shown association with a number of other malignant neoplasms, including renal carcinomas. In 2013, Wen et al. reported simultaneous renal clear cell carcinoma and GIST in a 65-year-old man who presented with abdominal discomfort, anorexia, weight loss, and weakness [14]. Dasanu et al. also reported a case of GIST with bilateral papillary RCCs [15]. To our knowledge, there has been no reported case in the English literature describing a patient with synchronous GIST and translocation-associated RCC.

Historically, RCCs have been defined by their histologic features. However, there exists a subset of renal carcinomas that are defined instead by their genetic make-up. In particular, this subset of RCCs is characterized by mutations involving chromosome Xp11 that lead to fusions of the* TFE3* transcription factor gene with various partner genes.

Renal carcinomas associated with Xp11.2 translocations were described as an entity distinct from other RCCs in 2004 by the World Health Organization [16]. They are relatively rare tumors that predominantly affect children and young adults and comprise between 26 and 40 percent of all pediatric RCCs [17–20]. Although the vast majority affects younger individuals, these tumors have been reported in older patients as well [21, 22]. In recent years, studies have suggested that this entity is more common in adults than was previously believed and make up from 1.6 to 5 percent of adult RCCs [23, 24].

Although Xp11.2 renal carcinomas are not defined by their histologic features—and in fact can show varied morphologies—they are often described to have a papillary architecture and to be comprised of polygonal cells with abundant clear to eosinophilic cytoplasm. These tumors can also demonstrate a more nested architecture, as well as cells with granular and eosinophilic cytoplasm. The* ASPL-TFE3* gene fusion variant, which is one of the more common variants, demonstrates characteristic features including cells with voluminous clear to eosinophilic cytoplasm as well as psammomatous calcifications [25], while the* PRCC-TFE3* variants are comprised of cells with less abundant cytoplasm, fewer psammoma bodies, and a more solid nested architecture [21].

The immunoprofile of these tumors differs from that of most RCCs, with no or only focal immunoreactivity for EMA, Cam 5.2, and vimentin. Strong nuclear immunoreactivity for TFE3 is the most distinctive immunohistochemical feature of these tumors [26]. The tumors also generally stain for renal cell carcinoma (RCC) marker antigen as well as CD10.

The differential diagnosis includes other renal carcinomas, such as clear cell RCC, papillary RCC, chromophobe RCC, and oncocytic renal carcinomas, as histopathologic features may overlap in these tumors. The clinical history can help in pediatric cases given that conventional RCCs are not as common in younger age groups relative to translocation-associated RCCs. However, in adult patients, the morphologic overlap between some instances of these tumors may lead to misclassification unless immunohistochemical staining for TFE3 and epithelial markers, as well as close attention to subtle unusual features such as cells with voluminous cytoplasm and increased psammomatous calcifications, is performed.

Angiomyolipomas (AML), which are part of the perivascular epithelioid cell tumor (PEComa) group of neoplasms, may also enter the differential diagnosis because their epithelioid variants are composed of polygonal cells that are arranged in a nested or focally acinar growth pattern. Furthermore, there have been some case reports of PEComas, including a renal epithelioid AML, with positive TFE3 immunolabeling and* TFE3* gene fusions [27]. However, AMLs will generally have some morphologic component of spindled cells, thick-walled vessels/vessels with perivascular hyalinization, or adipocytic cells, findings which can aid in their distinction from Xp11 RCCs. In addition to these morphologic attributes, PEComas will also lack immunoreactivity for epithelial or renal tubular markers and S100 and will be positive for HMB45 and Melan A.

Also in the differential with Xp11 translocation-associated RCC is renal carcinoma with t(6;11)(p21;q12) translocation. The latter appears to be less frequent, and it is characterized by a biphasic picture comprising large cells and small cells. The large cells contain clear to eosinophilic cytoplasm, distinct cell membranes, and vesicular nuclei with prominent nucleoli. The smaller cells demonstrate small nuclei with dense chromatin and scant cytoplasm, and they are often arranged around round cores of basement membrane-like material. Immunohistochemically, t(6;11) RCCs are diffusely nuclear positive for transcription factor EB (TFEB) and are usually positive for melanocytic markers such as Melan A and HMB-45 [28]. Our case focally demonstrated a seemingly biphasic picture comprising large cells and smaller cells that surrounded rounded hyaline-like structures, and there was positive immunolabeling for Melan A, prompting initial suspicion for a t(6;11) renal carcinoma. However, some cases of Xp11 RCC may also show the biphasic pattern with the small cells and hyaline material [29]. Furthermore, our RCC was positive for TFE3 and demonstrated the Xp11.2 translocation by cytogenetics, thus ruling out a t(6;11) carcinoma.

Finally with regard to the differential diagnosis, it is important to note that Xp11.2 translocations are not specific to the renal entity. Such translocations can also be seen in other neoplasms, including alveolar soft part sarcoma, a rare sarcoma with some morphologic features that superficially resemble those seen in some Xp11 RCCs [25].

Xp11 translocation-associated RCCs are defined by chromosomal translocations that result in fusion between the* TFE3* gene (on Xp11.2) and several other identified genes. These translocations can be detected by classical cytogenetics, fluorescent in situ hybridization (FISH), or polymerase chain reaction (PCR). One of the most common translocation variants is t(X;1)(p11.2;q21), which results in a* PRCC-TFE3* gene fusion [21]. Other identified translocations include t(X;17)(p11.2;q25) that results in an* ASPL-TFE3* gene fusion, t(X;1)(p11.2;p34) that results in a* PSF-TFE3* gene fusion, and inv(X)(p11.2;q12) that results in the fusion of* NonO* and* TFE3* genes [21, 25, 29]. Chromosome analysis in our case showed an abnormal female karyotype with an unbalanced rearrangement involving an (X;22) translocation at Xp11.2 and 22p11.2. To our knowledge, there has been no other documented case involving this specific rearrangement. The clinical and prognostic significance of this particular translocation is not clear, but our patient presented with advanced (Stage III) disease.

While cases in younger patients may be indolent even when diagnosed at an advanced stage, adult patients with Xp11.2 translocation renal carcinomas tend to present at an advanced stage and to have poor clinical outcomes. A 2007 study by Argani et al. of 28 cases showed that half of these patients presented with stage 4 disease and that lymph nodes were positive in 11 of 13 cases for which resections were performed [29]. A 2013 study by Zou et al. of 9 cases also supported the aggressiveness of at least some of these tumors, with almost half of their patients (4/9) presenting with stages 3-4 disease and 6 patients dying 10 months to 9 years following their operations [30].

The Xp11.2 translocation renal carcinoma in our patient was found incidentally as part of the work-up for a synchronous large gastric GIST. The majority of GISTs occur in the stomach, with about 54 percent occurring in the stomach, 32 percent in the small intestine, and rarer occurrences in the colon, rectum, and esophagus [31]. According to the Surveillance, Epidemiology, and End Results (SEER) database, GISTs account for about 2.2 percent of all malignant tumors, and estimates are that about 25 percent of gastric GISTs are clinically malignant [31, 32].

This patient developed two synchronous primary tumors. While the etiology of multiple tumors is complex and related to a variety of factors including environmental, genetic, and hormonal, the possible underlying relationship between GISTs and RCCs is an interesting concept. In Wen's article, the authors point out that both RCCs and GISTs, which are related to the receptor tyrosine kinase genes c-MET and c-KIT, are susceptible to treatment with sunitinib [14]. Additionally, it is believed that TFE3 mediates transcriptional upregulation of MET receptor tyrosine kinase [17]. Sunitinib is a multikinase inhibitor that inhibits vascular endothelial growth factor receptor, platelet-derived growth factor receptor alpha and beta, and c-KIT, as well as other kinases [33], and is an FDA approved drug for the treatment of RCCs and imatinib-resistant GISTs. Its effectiveness against both types of tumors may suggest that these tumors may have similar pathways of tumorigenesis. However, the association between these two tumors still needs to be further investigated.

## 4. Conclusions

In summary, we describe the first report to our knowledge of an adult patient with GIST and synchronous Xp11 translocation-associated RCC, the latter of which harbored a novel rearrangement involving an (X;22) translocation at Xp11.2 and 22p11.2. Although the clinical and prognostic significance of this specific translocation is unknown, the case fits with previously reported cases of adult Xp11 translocation renal tumors that demonstrate higher stage at diagnosis.

## References

[1] Nilsson B., Bümming P., Meis-Kindblom J. M. (2005). Gastrointestinal stromal tumors: the incidence, prevalence, clinical course, and prognostication in the preimatinib mesylate era—a population-based study in western Sweden. *Cancer*.

[2] Postow M. A., Robson M. E. (2012). Inherited gastrointestinal stromal tumor syndromes: mutations, clinical features, and therapeutic implications. *Clinical Sarcoma Research*.

[3] Qiao G.-B., Fang Y., Zeng W.-S., Peng L.-J., Huang W.-J. (2010). An unusual case of Carney triad with high level catecholamine-secreting but no existence of extra-adrenal paraganglioma. *Chinese Medical Journal*.

[4] Zhang L., Smyrk T. C., Young W. F., Stratakis C. A., Carney J. A. (2010). Gastric stromal tumors in Carney triad are different clinically, pathologically, and behaviorally from sporadic gastric gastrointestinal stromal tumors: findings in 104 cases. *The American Journal of Surgical Pathology*.

[5] Song H.-J., Kim K.-M., Dong I. C., Cheol K. P. (2009). Carney triad in an adult with aggressive behavior: the first case in Korea. *Yonsei Medical Journal*.

[6] Carney J. A. (2009). Carney triad: a syndrome featuring paraganglionic, adrenocortical, and possibly other endocrine tumors. *The Journal of Clinical Endocrinology & Metabolism*.

[7] Sawhney S. A., Chapman A. D., Carney J. A., Gomersall L. N., Dempsey O. J. (2009). Incomplete carney triad—a review of two cases. *QJM*.

[8] Qiao G.-B., Zeng W.-S., Peng L.-J. (2009). Multiple pulmonary chondromas in a young female patient: a component of carney triad. *Journal of Thoracic Oncology*.

[9] Alberto V. O., Kelleher D., Denholm R. B., Nutt M., Carney J. A. (2008). A calcified lung tumour and microcytic anaemia in a young woman: partial expression of the Carney triad. *Surgeon*.

[10] Robson M. E., Glogowski E., Sommer G. (2004). Pleomorphic characteristics of a germ-line KIT mutation in a large kindred with gastrointestinal stromal tumors, hyperpigmentation, and dysphagia. *Clinical Cancer Research*.

[11] Rodriguez F. J., Aubry M.-C., Tazelaar H. D., Slezak J., Aidan Carney J. (2007). Pulmonary chondroma: a tumor associated with carney triad and different from pulmonary hamartoma. *The American Journal of Surgical Pathology*.

[12] Jabbour S. A., Miller J. L. (1999). A case of the Carney triad. *Endocrine Practice*.

[13] Carney J. A. (1999). Gastric stromal sarcoma, pulmonary chondroma, and extra-adrenal paraganglioma (Carney triad): natural history, adrenocortical component, and possible familial occurrence. *Mayo Clinic Proceedings*.

[14] Wen J., Li H.-Z., Ji Z. G., Wei G.-Y., Shi B. B. (2013). Simultaneous renal clear cell carcinoma and gastrointestinal stromal tumor in one case. *Urology Annals*.

[15] Dasanu C. A., Jethava A., Ali S., Codreanu I. (2013). Gastrointestinal stromal tumor of small intestine and synchronous bilateral papillary renal cell carcinoma. *Connecticut Medicine*.

[16] Argani P., Ladanyi M., Eble J. N., Sauter G., Epstein J. I., Sesterhenn I. A. (2004). Renal carcinomas associated with Xp11.2 translocations/TFE3 gene fusions. *WHO Classification of Tumours of the Urinary System and Male Genital Organs*.

[17] Klaassen Z., Tatem A., Burnette J. O., Donohoe J. M., Terris M. K. (2012). Adult Xp11 translocation associated renal cell carcinoma: time to recognize. *Urology*.

[18] Ramphal R., Pappo A., Zielenska M., Grant R., Ngan B.-Y. (2006). Pediatric renal cell carcinoma: clinical, pathologic, and molecular abnormalities associated with the members of the MiTTranscription factor family. *American Journal of Clinical Pathology*.

[19] Wu A., Kunju L. P., Cheng L., Shah R. B. (2008). Renal cell carcinoma in children and young adults: analysis of clinicopathological, immunohistochemical and molecular characteristics with an emphasis on the spectrum of Xp11.2 translocation-associated and unusual clear cell subtypes. *Histopathology*.

[20] Argani P., Laé M., Ballard E. T. (2006). Translocation carcinomas of the kidney after chemotherapy in childhood. *Journal of Clinical Oncology*.

[21] Argani P., Antonescu C. R., Couturier J. (2002). PRCC-TFE3 renal carcinomas: morphologic, immunohistochemical, ultrastructural, and molecular analysis of an entity associated with the t(X;1)(p11.2;q21). *American Journal of Surgical Pathology*.

[22] Arnoux V., Long J.-A., Fiard G. (2012). Xp11.2 translocation renal carcinoma in adults over 50 years of age: about four cases. *Progres en Urologie*.

[23] Komai Y., Fujiwara M., Fujii Y. (2009). Adult Xp11 translocation renal cell carcinoma diagnosed by cytogenetics and immunohistochemistry. *Clinical Cancer Research*.

[24] Zhong M., De Angelo P., Osborne L. (2012). Translocation renal cell carcinomas in adults: a single-institution experience. *The American Journal of Surgical Pathology*.

[25] Argani P., Antonescu C. R., Illei P. B. (2001). Primary renal neoplasms with the ASPL-TFE3 gene fusion of alveolar soft part sarcoma: a distinctive tumor entity previously included among renal cell carcinomas of children and adolescents. *American Journal of Pathology*.

[26] Argani P., Lal P., Hutchinson B., Lui M. Y., Reuter V. E., Ladanyi M. (2003). Aberrant nuclear immunoreactivity for TFE3 in neoplasms with TFE3 gene fusions: a sensitive and specific immunohistochemical assay. *The American Journal of Surgical Pathology*.

[27] Ohe C., Kuroda N., Hes O. (2012). A renal epithelioid angiomyolipoma/perivascular epithelioid cell tumor with *TFE3* gene break visualized by FISH. *Medical Molecular Morphology*.

[28] Kuroda N., Tanaka A., Sasaki N. (2013). Review of renal carcinoma with t(6;11)(p21;q12) with focus on clinical and pathobiological aspects. *Histology and Histopathology*.

[29] Argani P., Olgac S., Tickoo S. K. (2007). Xp11 Translocation renal cell carcinoma in adults: expanded clinical, pathologic, and genetic spectrum. *American Journal of Surgical Pathology*.

[30] Zou H., Kang X., Pang L. J. (2013). Xp11 translocation renal cell carcinoma in adults: a clinicopathological and comparative genomic hybridization study. *International Journal of Clinical and Experimental Pathology*.

[31] Miettinen M. M., Corless C. L., Debiec-Rychter M., Fletcher C. D. M., Bridge J. A., Hogendoorn P. C. W., Mertens F. (2013). Gastrointestinal stromal tumors. *WHO Classification of Tumours of Soft Tissue and Bone*.

[32] Thomas R. M., Sobin L. H. (1995). Gastrointestinal cancer. *Cancer*.

[33] Imbulgoda A., Heng D. Y. C., Kollmannsberger C. (2014). Sunitinib in the treatment of advanced solid tumors. *Small Molecules in Oncology*.

